# Patient-Derived Scaffolds of Colorectal Cancer Metastases as an Organotypic 3D Model of the Liver Metastatic Microenvironment

**DOI:** 10.3390/cancers12020364

**Published:** 2020-02-05

**Authors:** Edoardo D’Angelo, Dipa Natarajan, Francesca Sensi, Omolola Ajayi, Matteo Fassan, Enzo Mammano, Pierluigi Pilati, Piero Pavan, Silvia Bresolin, Melissa Preziosi, Rosa Miquel, Yoh Zen, Shilpa Chokshi, Krishna Menon, Nigel Heaton, Gaya Spolverato, Martina Piccoli, Roger Williams, Luca Urbani, Marco Agostini

**Affiliations:** 1 First Surgical Clinic, Department of Surgery, Oncology and Gastroenterology, University of Padova, 35128 Padova, Italy; edoardo.dangelo@unipd.it (E.D.); gaya.spolverato@gmail.com (G.S.); 2 LIFELAB Program, Consorzio per la Ricerca Sanitaria-CORIS, Veneto Region, 35128 Padova, Italy; 3 Institute of Hepatology, Foundation for Liver Research, London SE5 9NT, UK; dipa.natarajan@researchinliver.org.uk (D.N.); l.ajayi@researchinliver.org.uk (O.A.); s.chokshi@researchinliver.org.uk (S.C.); r.williams@researchinliver.org.uk (R.W.); 4 Faculty of Life Sciences & Medicine, King’s College London, London WC2R 2LS, UK; 5 Fondazione Istituto di Ricerca Pediatrica Città della Speranza, 35127 Padova, Italy; sensifrancy@gmail.com (F.S.); piero.pavan@dicea.unipd.it (P.P.); silvi.bresolin@gmail.com (S.B.); m.piccoli@irpcds.org (M.P.); 6 Department of Women’s and Children’s Health, University of Padova, 35128 Padova, Italy; 7 Department of Medicine, University of Padova, 35128 Padova, Italy; matteo.fassan@gmail.com; 8 Unit of Surgical Oncology of the Esophagus and Digestive Tract, Veneto Institute of Oncology IOV-IRCCS, 35128 Padova, Italy; enzo.mammano@sanita.padova.it (E.M.); pierluigi.pilati@iov.veneto.it (P.P.); 9 Department of Industrial Engineering, University of Padova, 35131 Padova, Italy; 10 Laboratory of Onco-Hematology, Department of Women’s and Children’s Health, University of Padova, 35128 Padova, Italy; 11 Institute of Liver Studies, King’s College London, London SE5 9RS, UK; melissa.preziosi1@nhs.net (M.P.); krishna.menon@nhs.net (K.M.); nigel.heaton@nhs.net (N.H.); 12 Liver Histopathology Laboratory, Institute of Liver Studies, King’s College London, London SE5 9RS, UK; rosa.miquel@nhs.net (R.M.); yoh.zen@nhs.net (Y.Z.)

**Keywords:** microenvironment, extracellular matrix, disease modeling, 3D culture model, liver metastasis, decellularization, drug test, response to treatment

## Abstract

The liver is the most common site for colorectal cancer (CRC) metastasis and there is an urgent need for new tissue culture models to study colorectal cancer liver metastasis (CRLM) as current models do not mimic the biological, biochemical, and structural characteristics of the metastatic microenvironment. Decellularization provides a novel approach for the study of the cancer extracellular matrix (ECM) as decellularized scaffolds retain tissue-specific features and biological properties. In the present study, we created a 3D model of CRC and matched CRLM using patient-derived decellularized ECM scaffolds seeded with the HT-29 CRC cell line. Here, we show an increased HT-29 cell proliferation and migration capability when cultured in cancer-derived scaffolds compared to same-patient healthy colon and liver tissues. HT-29 cells cultured in CRLM scaffolds also displayed an indication of epithelial-mesenchymal transition (EMT), with a loss of E-cadherin and increased Vimentin expression. EMT was confirmed by gene expression profiling, with the most represented biological processes in CRLM-seeded scaffolds involving demethylation, deacetylation, a cellular response to stress metabolic processes, and a response to the oxygen level and starvation. HT-29 cells cultured in cancer-specific 3D microenvironments showed a reduced response to treatment with 5-fluorouracil and 5-fluorouracil combined with Irinotecan when used at a standard IC_50_ (as determined in the 2D culture). Our 3D culture system with patient-derived tissue-specific decellularized ECM better recapitulates the metastatic microenvironment compared to conventional 2D culture conditions and represents a relevant approach for the study of CRLM progression and assessing the response to chemotherapy agents.

## 1. Introduction 

Colorectal cancer (CRC) is the third most common cancer worldwide and is ranked as the second leading cause of cancer-related deaths [1]. The liver is the most common site of CRC metastasis as the majority of intestinal mesenteric drainage enters the hepatic portal venous system [2] and, at the time of diagnosis, up to 25% of patients have a synchronous liver metastasis. In addition, after resection of the primary tumor, approximately 25% of patients will be diagnosed with liver metastasis within the next 3 years [3]. Any hypothesis to explain why the liver is a main target of CRC metastasis cannot be restricted to the fact that the liver represents the first capillary bed to which disseminated CRC cells can migrate. According to the ‘seed and soil’ hypothesis, some distant organs provide a more favorable micro-environment for the colonization of cancer cells than others [4]. Although the genetic characteristics of tumor cells are essential for conferring a metastatic-prone phenotype, crucial micro-environmental cues cannot be disregarded in cancer cell colonization and metastasis.

Currently, most liver colonization studies are performed with xenograft models, which cannot be used to monitor the dynamic process of metastasis and are costly and time consuming. Very few studies describe tissue culture models which are able to mimic the metastatic environment [5] and, in regard to the seed and soil hypothesis, it is difficult to study the mechanism behind both the micro-environment and the cancer cell at the same time. Although current technologies allow isolation of the patient’s metastatic cells—the “seed”—these are then studied in a microenvironment—the “soil”—which is highly different from the pathological state. However, a recent study described a physiologically-relevant tissue culture model to investigate the liver colonization of CRC using tissue samples from a rat model [6]. The authors showed that, in a 2D culture system, extracellular matrix (ECM) from specific tissues (e.g., liver and lung) was better able to mimic the metastatic environment than conventional plastic plates or those coated with collagen or Matrigel. 

Three-dimensional (3D) culture models have been widely used to investigate CRC pathogenesis, progression, and treatment responses [7]. The composition and enrichment of ECM molecules are distinct from one organ to another and the metastatic niche is a complex environment that co-evolves with cancer cells. High-throughput proteomics was used to analyze the expression profiles of ECM proteins in healthy colon, primary CRC, healthy liver (HL), and colorectal cancer liver metastasis (CRLM) [8], and a “matrisome” was identified for each tissue. Importantly, the ECM composition of CRLM more closely resembled the ECM of primary CRC than that of a normal liver. 

Progress in decellularization techniques provides novel approaches for isolating patient-derived ECM [9]. Decellularization is successfully used in tissue engineering for organ reconstruction and has been applied to the study of many solid tumors, including CRC [10,11,12,13,14,15]. We recently described a protocol for the decellularization of healthy colon (HC) and CRC tissue, in order to obtain matrices with preserved biological and ultrastructural properties [16]. In the present study, we aim to develop an ex vivo 3D model of CRLM and matched CRC using decellularized patient-derived liver matrix and CRC cell lines, but not to follow the metastatic mechanism in real-time or to in vitro model the metastatic process. We provide an in-depth characterization of the properties and structure of the 3D CRLM model, demonstrating that it recapitulates features of the tissue-specific microenvironment of CRLM in vitro.

## 2. Results

### 2.1. Decellularization and Characterization of 3D Patient-Derived Scaffolds 

**Figure 1:**
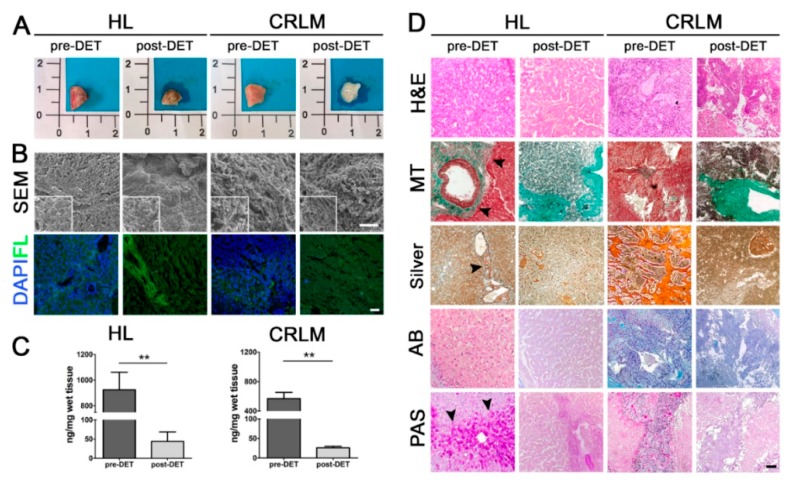
Decellularization and characterization of organotypic patient-derived decellularized scaffolds of colorectal cancer liver metastases. (**A**) Gross appearance of healthy liver (HL) and colorectal cancer liver metastasis (CRLM) tissue samples, before (pre-detergent-enzymatic treatment (DET)) and after (post-DET) two decellularization cycles. (**B**) Scanning electron microscope and immunofluorescence images of fresh and decellularized biopsies of HL and CRLM. DAPI: 4’,6-diamidin-2-fenilindolo (blue); FL: autofluorescence of tissue samples to show tissue preservation (green). Scale bar: 100 µm. (**C**) DNA quantification of tissue samples before and after decellularization; *n* = 3. ** *p*-value < 0.01 by Student’s *t*-test. (**D**) Histological staining of HL and CRLM samples pre-DET and post-DET.; H&E: Haematoxylin and Eosin; MT: Masson’s Trichrome, with fibrillar collagens indicated by black arrow heads; Silver: Silver stains, with reticular collagens indicated by black arrow heads; AB: Alcian blue; PAS: Periodic Acid-Schiff, with glycogen indicated by black arrow heads. Scale bar: 100 µm.

After the detergent-enzymatic treatment (DET), both heathy liver (HL) and CRLM maintained a phenotypical appearance similar to the pre-DET counterpart, with no obvious degradation or disintegration. Post-DET CRLM tissue showed an increased translucency compared to fresh tissue, while post-DET HL maintained a brown color (Figure 1A). SEM analysis and DAPI staining confirmed successful cellular depletion and ultrastructure maintenance after the decellularization process (Figure 1B). The quantification of DNA after each DET cycle confirmed that HL and CRLM samples were successfully decellularized after two DET cycles, with a reduction of DNA content of 95.3% (*p*-value = 0.0032) and 95.42% (*p*-value = 0.0032), respectively, compared to original pre-DET tissues (Figure 1C). The absence of cellular content in post-DET HL and CRLM tissues was confirmed with H&E staining (Figure 1D).

Immunohistochemical analysis showed that the expression and distribution of key ECM components, such as collagen and glycosaminoglycans, were maintained in post-DET HL and CRLM tissues. MT and Van Gieson’s staining revealed the preservation of collagen structures through the portal tracts and vessel walls after decellularization, but underlined the absence of linear collagen fibers in the parenchymal space of both pre- and post-DET CRLM tissues compared with HL tissue (Figure 1D and Supplementary Figure S1A), as was also evident with anti-Collagen IV immunostaining (Supplementary Figure S1B). In contrast, the Silver stain for reticulin fibers highlighted a hyperplastic expansion of the space of Disse commonly observed in regenerative and neoplastic conditions (Figure 1D); characteristics that were maintained in decellularized samples. AB showed a lower presence of acid mucins, stained in blue, in both pre- and post-DET HL tissues, and a stronger signal in both pre- and post-DET CRLM samples (Figure 1D). PAS staining of neutral mucins and glycogen in purple-magenta, showed a reduced glycogen content in post-DET HL tissues compared to the pre-DET counterpart, highlighting that this component is sensitive to the DET. In both pre- and post-DET CRLM samples, the over-production of acid mucins highlighted by the AB stain and generally observed in CRC pathological evolution, was absent in HL. Taken together, the data confirm that the decellularization method was able to remove the resident cells whilst overall preserving the biochemical and structural properties of both healthy and metastatic liver ECM. 

### 2.2. Colonization and Proliferation of CRC Cells in Patient-Derived Scaffolds

**Figure 2:**
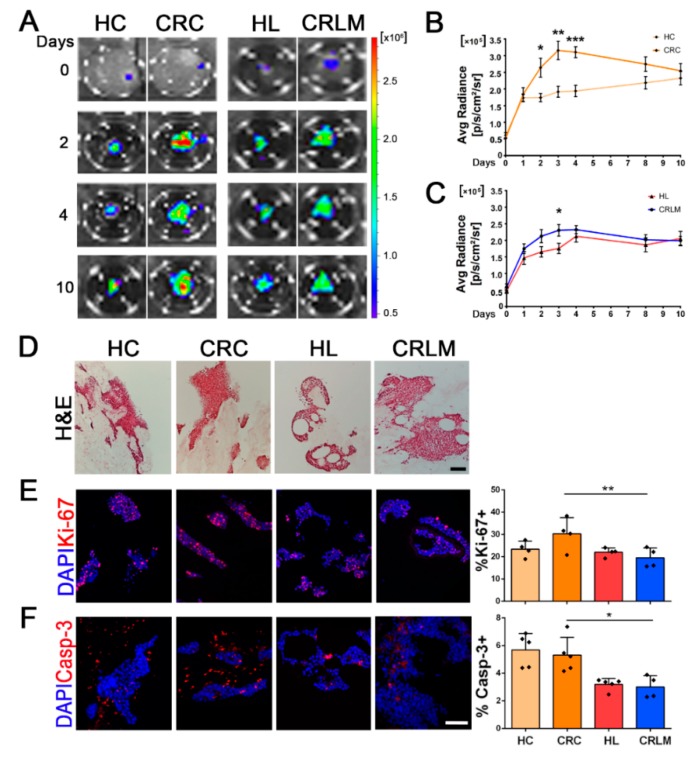
Characterization of recellularized CRC and CRLM scaffolds using IVIS and immunofluorescence. (**A**) Representative bioluminescence pictures show the proliferation rate of HT-29 ZsGreen/Luc^+^ cells seeded in healthy (healthy colon (HC) and HL) and neoplastic decellularized scaffolds (colorectal cancer (CRC) and CRLM) from day 0 to day 10. (**B**) Line graphs summarize the proliferation rate of HT-29-recellularized CRC vs. HC (HC, *n* = 4; CRC, *n* = 4) and (**C**) CRLM vs. HL (HL, *n* = 4; CRLM, *n* = 5). (**D**) Representative H&E images of 3D patient-derived scaffolds 10 days after recellularization with HT-29 cells. Immunofluorescent images and quantifications at day 10 of (**E**) Ki-67^+^ and (**F**) activated Caspase-3^+^; nuclei were counterstained with DAPI (blue). Scale bar: 50 µm. For line graphs, * *p*-value < 0.05; ** *p*-value < 0.01; and *** *p*-value < 0.001 by Student’s *t*-test. For graph bars, * *p*-value < 0.05 and ** *p*-value < 0.01 by a one-way ANOVA test following Tukey’s Multiple Comparison Test.

To test whether patient-derived decellularized scaffolds could support cell viability in a tissue-specific fashion, we cultured Luc-ZsGreen^+^HT-29 cells (Supplementary Figure S2) in CRC and CRLM scaffolds, using their respective healthy samples as controls. HT-29 cells were equally able to adhere to and colonize all four types of scaffolds, as demonstrated by longitudinal bioluminescence analysis, although significantly different metabolic activities were detected among tissue samples (Figure 2A). In CRC scaffolds, HT-29 cells demonstrated a significantly higher cell viability compared to HC in the first 4 days of culture, (day 2 *p*-value = 0.011; day 3 *p*-value = 0.0017; day 4 *p*-value = 0.00023; respectively) (Figure 2B). Cells seeded in HL and CRLM scaffolds showed an initial difference in the viability rate, which was significantly higher in CRLM- compared to HL-seeded scaffolds at day 3 (*p*-value = 0.034), and subsequently, a similar trend was exhibited for up to 10 days of culture (Figure 2C). The ability of cells to adhere to and grow in the decellularized scaffolds was confirmed by staining sections with H&E at day 10 of culture. Clusters of cells of different sizes were identified within the scaffolds (Figure 2D). Compared to the 2D conventional culture (Supplementary Figure S3), proliferation was reduced in 3D culture conditions at day 10 of culture (end point), as highlighted by immunofluorescence staining for the marker Ki-67 (Figure 2E). Among the seeded scaffolds, a higher relative number of Ki-67^+^ cells was observed in CRC scaffolds compared to all other samples (*p*-value = 0.044), with no significant difference between HC, HL, and CRLM scaffolds, strongly supporting the bioluminescence data. Immunofluorescence analysis for activated Caspase-3 showed a significantly higher apoptosis rate in both colon-derived scaffolds (HC and CRC) compared to liver-derived samples (both HL and CRLM) (*p*-value = 0.016) (Figure 2F). A similar trend, although not significant, was observed in scaffolds repopulated with a different CRC cell line. HCT-116-repopulated CRC scaffolds showed an increased percentage of Ki-67-positive cells compared to HL and CRLM scaffolds. Relative numbers of Caspase-3-positive cells were comparable between HT-29 and HCT-116 repopulated scaffolds (respectively, Figure 2F and Supplementary Figure S4A,B).

### 2.3. Migration of CRC Cells in Patient-Derived CRC and CRLM Scaffolds 

**Figure 3:**
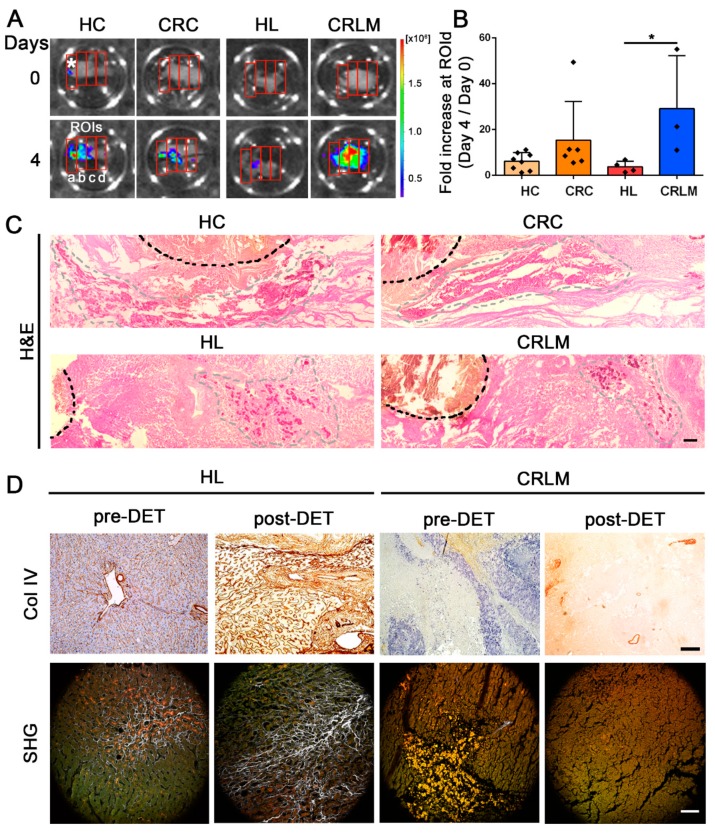
Migration analysis of recellularized CRC and CRLM scaffolds and collagen distribution. (**A**) Representative bioluminescence pictures show the migration rate of HT-29 cells seeded in healthy (HC and HL) and neoplastic decellularized scaffolds (CRC and CRLM) from day 0 to day 4. Asterisk: HT-29 cell injection point. a, b, c, d, and red rectangles: regions of interest (ROIs). (**B**) Bar graph summarizes the migration rate of HT-29-recellularized scaffolds (HC, *n* = 3; CRC, *n* = 3; HL, *n* = 4; CRLM, *n* = 3). Cell migration was calculated as the fold increase in ROI d between day 0 and day 4. (**C**) Representative H&E images at day 4 show the injection site of HT-29 cells in each scaffold (black dotted line) and the differential cell migration ability (migration front highlighted with a gray dotted line). Scale bar: 100 µm. (**D**) Top: Collagen IV (Col IV) immunohistochemistry of normal (pre-DET) and decellularized (post-DET) tissue samples of HL and CRLM. Scale bar: 100 µm. Bottom: second-harmonic generation images (SHG) of pre-DET and post-DET tissue samples of HL and CRLM. Collagen I in SHG images is highlighted in white, nuclei in light orange, and lipofuscin granules in dark orange. Scale bar: 100 µm. * *p*-value < 0.05; by a one-way ANOVA test following Tukey’s Multiple Comparison Test.

An investigation of cell migration in 3D scaffolds showed that HT-29 cells injected into the left side of the scaffolds (ROI a) and cultured for 4 days, migrated to the right-end of the scaffold (ROI d) (Figure 3A–C). HT-29 cells seeded in healthy tissue scaffolds (both HC and HL) showed a similar lower migration ability compared to the tumor-derived scaffolds. CRLM-recellularized scaffolds exhibited a significantly higher migration potential compared to HL (*p*-value = 0.018) and a similar trend compared to CRC scaffolds (Figure 3B). Cells in HC scaffolds displayed a peri-injection site (highlighted with a black line) distribution, while a more marked migration aptitude was observed in CRLM- and CRC-seeded samples (Figure 3C). Since a fine balance between collagen fiber linearization and reticular collagen abundance is crucial for metastatic cell extravasation and migration [17,18], we also analyzed the parenchyma in CRC and CRLM scaffolds. We detected a low content of linear collagen, namely collagen IV (Figure 3D), but a high density of reticular fibers and other ECM-associated protein components in the CRLM stroma, as highlighted by H&E staining (Figure 3C) and SHG imaging (Figure 3D).

### 2.4. CRC Cell Phenotype and Epithelia-to-Mesenchymal Induction Analysis

**Figure 4:**
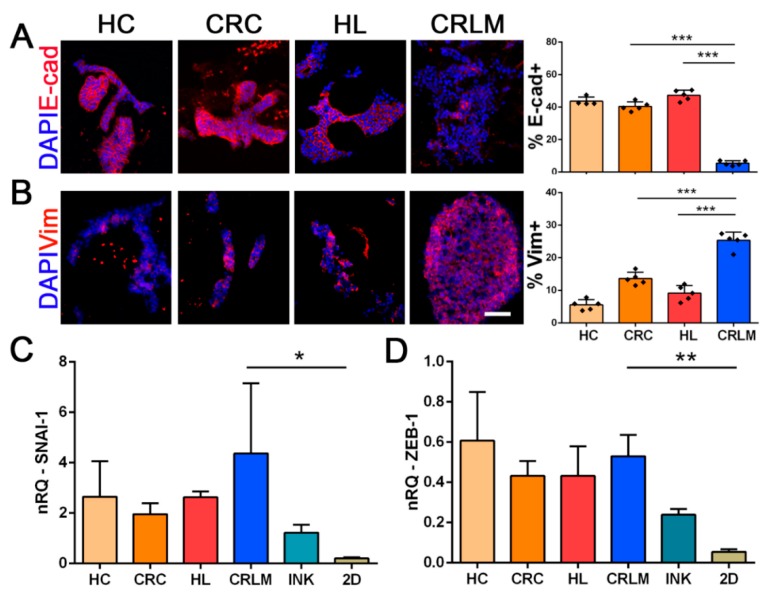
Phenotypic and molecular comparison of EMT markers between recellularized scaffolds and HT-29 cells cultured in 2D. Immunofluorescence images and quantifications at day 10 of (**A**) E-cadherin^+^ and (**B**) Vimentin^+^; nuclei were counterstained with DAPI (blue). (**C**) and (**D**) Gene expression level, after 10 days of culture, of *SNAI-1* and *ZEB-1* HT-29 ZsGreen/Luc^+^ cells, respectively, seeded in HC, CRC, HL, CRLM, 3D-bioprintable ink (INK), and a conventional plastic plate (2D). Scale bar: 50 µm. Nrq = normalized relative quantity for qRT-PCR data. Graph bars, * *p*-value < 0.05; ** *p*-value < 0.01; and *** *p*-value < 0.001 by a one-way ANOVA test following Tukey’s Multiple Comparison Test.

Given the different cell behavior among the 3D cultures, we investigated the EMT profile of tumor cells. Immunofluorescence analysis showed a significant reduction of E-cadherin expression when HT-29 cells were cultured in CRLM scaffolds compared to HL and CRC samples (*p*-value < 0.001 and *p*-value < 0.0001, respectively) (Figure 4A), whereas they exhibited a significantly increased expression of Vimentin only when cultured in CRLM scaffolds compared to CRC and both healthy scaffolds (*p*-value < 0.001 vs. CRC; *p*-value < 0.001 vs. HL and *p*-value = 0.001 vs. HC) (Figure 4B). In HCT-116-repopulated scaffolds, E-cadherin and Vimentin proteins showed a homogenous abundance in all scaffolds. At a transcriptomic level, no significant differences were observed in the gene expression of E-cadherin and Vimentin (Supplementary Figure S4C,D). In addition, HT-29 cells displayed significant *SNAI-1* and *ZEB-1* gene up-regulation in CRLM scaffolds compared with 2D culture conditions (respectively, *p*-value < 0.05 and *p*-value < 0.01) (Figure 4C,D). When cells were cultured in 3D conditions but without the presence of ECM (3D-bioprintable ink), they showed an intermediate pattern of *SNAI-1* and *ZEB-1* gene expression, between what was detected in 3D patient-derived scaffolds and conventional 2D cultures (Figure 4C,D and Supplementary Figure S5). When comparing HT-29 cells cultured in the 3D-bioink conditions with conventional 2D cultures, the expression level of E-cadherin was slightly diminished in the 3D condition, while Vimentin was completely absent in 3D-bioink and at a low basal level in 2D cultures (Supplementary Figures S3 and S5C). In HCT-116 cell-repopulated scaffolds, we observed no difference in the expression of the *SNAI-1* gene among recellularized 3D scaffolds, although it was significantly higher than in 2D cell cultures (Supplementary Figure S4E). We also observed up-regulation of the *ZEB-1* gene only when cells were cultured in CRLM scaffolds (CRLM vs. 2D, *p*-value < 0.001 and CRLM vs. HL, *p*-value < 0.05) (Supplementary Figure S4F). 

### 2.5. Gene Expression Profile Analysis in Recellularized Patient-Derived HL and CRLM Scaffolds

**Figure 5:**
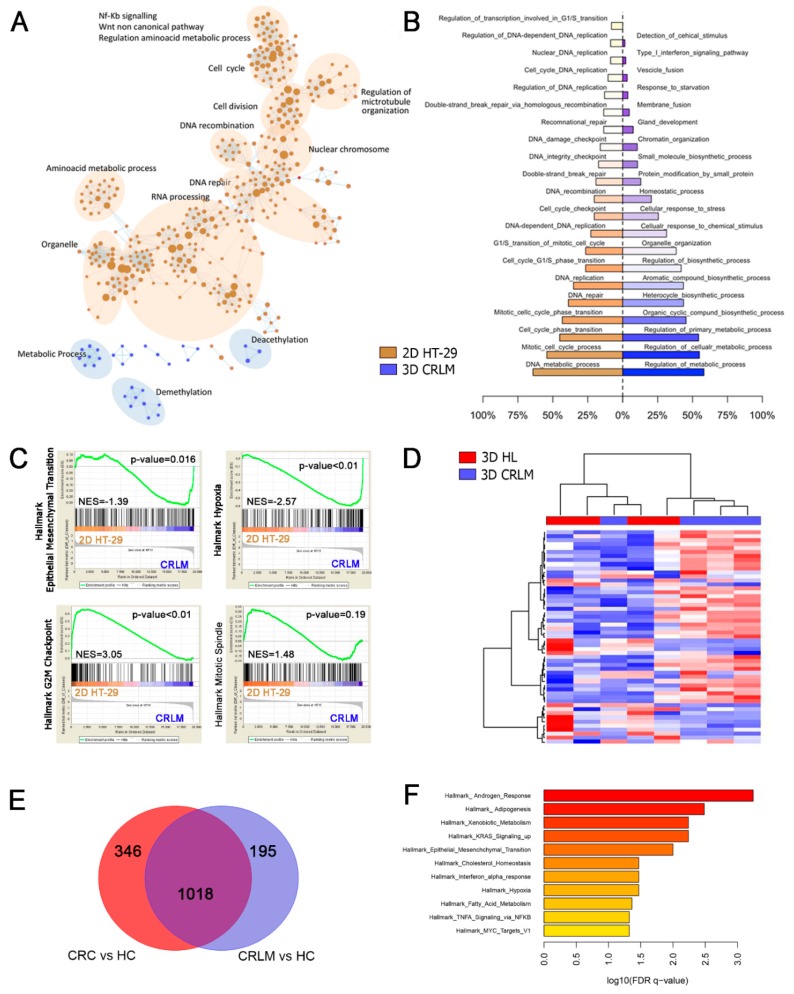
Global gene expression analysis of HT-29 cells in 2D and in HL and CRLM 3D culture models. (**A**) Enriched biological process in recellularized CRLM scaffolds (*n* = 4), after 10 days of growth (blue) and in HT-29 cells grown in 2D (brown) using gene ontology MSigDB. (**B**) Parallel analysis of the most enriched biological processes in recellularized CRLM scaffolds, after 10 days of growth (blue) and in HT-29 cells grown in 2D (brown), using the SAM identified differently expressed genes. (**C**) Comparison of the differently expressed genes between recellularized CRLM scaffolds and HT-29 cells grown in 2D with an a-priori defined gene-set called Hallmark. Epithelial_Mesenchymal_Transition and Hypoxia are biological pathways enriched in recellularized CRLM scaffolds, while G2M_Checkpoint is enriched in HT-29 cells grown in 2D. The horizontal bar displaying a graded color from brown (left) to blue (right) represents the genes list ranked from high expression in the HT-29 cells grown in the 2D subset indicated on the left to high expression in the recellularized CRLM scaffolds subset indicated on the right. The vertical black lines (‘bar code’) represent the projection onto the ranked genes list of the individual genes. The curve in green corresponds to the calculation of the enrichment score (ES). The bold horizontal line indicates the 0 value for the ES. The more the green ES curve is shifted to the upper left of the graph, the more the gene set is enriched in the HT-29 cells grown in the 2D subset. Conversely, the more the green ES curve is shifted to the lower right of the graph, the more the gene set is enriched in the recellularized CRLM scaffolds subset. Nominal p-values are indicated in the upper right corner of each plot, and Normalized Enrichment Scores (NES) are indicated in the middle-left of each plot. (**D**) Supervised hierarchical clustering analysis of recellularized HL-repopulated scaffolds (red) in comparison with CRLM-repopulated scaffolds (blue) using the 53 differently expressed genes obtained with Limma analysis. (**E**) SAM analysis of differently expressed genes between primary CRC or CRLM and healthy colon mucosa from CRLM patients. (**F**) Biological pathways enriched in the comparison of differently expressed genes between primary CRC or CRLM and HC from CRLM patients, with an a-priori-defined gene-set called Hallmark.

The transcriptomic profiles of each 3D subtype were compared with HT-29 cells grown in 2D. Unsupervised hierarchical clustering analysis revealed that the gene expression signature of recellularized CRLM scaffolds was more comparable to recellularized HL scaffolds, than to HT-29 cells grown in 2D (Supplementary Figure S6A). The high degree of separation between HT-29 grown in 2D versus both the CRLM- and HL-recellularized scaffold was also shown in a supervised SAM analysis. A total of 897 differently expressed genes (DEG) were recorded between recellularized CRLM scaffolds and HT-29 cells cultured in 2D, while a total of 449 DEG were observed between recellularized HL scaffolds and HT-29 cells cultured in 2D. By comparing the DEG between recellularized CRLM and HL scaffolds vs. HT-29 in 2D cultures, 475 were shown to be CRLM-exclusive, 27 were HL-exclusive, and 422 were shared between both recellularized CRLM and HL (Supplementary Figure S6B). Gene set enrichment analysis (GSEA) in recellularized CRLM scaffolds compared to HT-29 grown in 2D (using gene ontology MSigDB), revealed that up-regulated genes involved in the demethylation, deacetylation, and metabolic process belong to the most enriched biological pathways in repopulated CRLM scaffolds. On the other hand, the gene set up-regulated in HT-29 grown in 2D referred to RNA processing, DNA repair, DNA recombination, cell division, the cell cycle, and the amino acid metabolic process (Figure 5A). The cellular response to stress, response to the oxygen level, negative regulation of the cellular process, and response to starvation were the most enriched biological processes in repopulated CRLM scaffolds (Figure 5B) among the SAM-identified DEG. The same analysis highlighted that the regulation of transcription involved in the G1/S transition of mitosis, DNA replication, and replication initiation were the most enriched biological processes in HT-29 grown in 2D (Figure 5B). Comparing the DEG between recellularized CRLM scaffolds vs. HT-29 grown in 2D with a series of a-priori-defined gene-sets, called Hallmark, we obtained concordant results with the “HYPOXIA” pathway, enriched in recellularized CRLM, while the “G2M_CHECKPOINT” and “MITOTIC SPINDLE” biological processes were enriched in HT-29 grown in 2D. The overall “EPITHELIAL_MESENCHYMAL_TRANSITION” biological process was enriched in recellularized CRLM scaffolds (Figure 5C), supporting the data obtained in the phenotype characterization and migration test of HT-29 seeded in CRLM scaffolds. The list of DEG between recellularized CRLM scaffolds vs. HT-29 in 2D that belong to the “EPITHELIAL_MESENCHYMAL_TRANSITION” sub-group of Hallmark dataset can be found in Supplementary Table S1. In order to verify that HT-29 cells were affected in an organotypic manner, we compared the transcriptomic profiles of recellularized CRLM scaffolds and recellularized HL scaffolds. A total of 53 genes were differently expressed between recellularized CRLM and HL scaffolds. A supervised SAM analysis showed a high degree of separation between recellularized CRLM scaffolds and recellularized HL scaffolds, with only one mis-classification for both recellularized scaffold types (Figure 5D). Additionally, as summarized in Supplementary Table S2, we identified that 10 out of 53 DEG were actively involved in tumor invasion, migration, and EMT induction. Finally, in order to verify whether the DEG identified in our 3D repopulated CRLM scaffolds were relevant in CRLM patients, we applied GSEA analysis to the gene expression signature of CRC and CRLM primary tissue samples obtained from the GEO publicly available GSE62321 dataset. Data intersection between DEG genes obtained from the comparison of HC vs. primary CRC and HC vs. CRLM, identified 1018 common genes (Figure 5E). GSEA analysis confirmed that “EPITHELIAL_MESENCHYMAL_TRANSITION” and “HYPOXIA” biological processes were enriched in pathological tissue samples, in line with data obtained from HT-29 cells cultured in CRLM scaffolds (Figure 5F).

### 2.6. Therapeutic Response of 3D Patient-Derived Scaffolds to Different Chemotherapy Treatment Regimens

**Figure 6:**
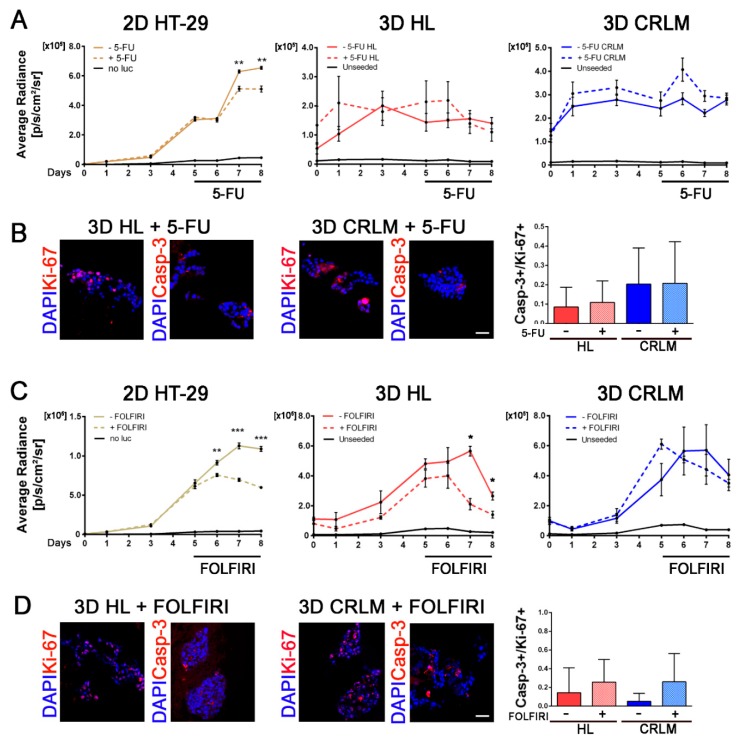
Chemotherapy treatment of recellularized HL and CRLM scaffolds and HT-29 cultured in 2D. (**A**) Cell viability measured in response to treatment with 5-FU. HT-29 cells were cultured in 2D (brown, +5-FU dotted line/-5-FU continuous line; *n* = 3 replicates) or in HL (*n* = 6) and CRLM (*n* = 6) scaffolds (respectively, red and blue, +5-FU dotted line/-5-FU continuous line) for 5 days and then exposed to 5-FU (1µM) for 3 days. Cultures without the addition of 5-FU or D-Luciferin (black), or unseeded scaffolds (black), were used as controls. (**B**) Representative immunofluorescent images and quantifications at day 8 of HT-29-recellularized scaffolds treated with 5-FU. Ki-67^+^ (Red) and activated Caspase-3^+^ (Red) cells; nuclei were counterstained with DAPI (blue). (**C**) Cell viability measured in response to treatment with FOLFIRI. HT-29 cells were cultured in 2D (brown, +FOLFIRI dotted line/-FOLFIRI continuous line; *n* = 3 replicates) or in HL (*n* = 9) and CRLM (*n* = 9) scaffolds (respectively, red and blue, +FOLFIRI continuous line/-FOLFIRI dotted line) for 5 days and then exposed to FOLFIRI (1 µM) for 3 d. Cultures without the addition of FOLFIRI or D-Luciferin (black), or unseeded scaffolds (black), were used as controls. (**D**) Representative immunofluorescent images and quantifications at day 8 of HT-29-recellularized scaffolds treated with FOLFIRI. Ki-67^+^ (Red) and activated Caspase-3^+^ (Red) cells; nuclei were counterstained with DAPI (blue). Scale bar: 50 µm. For line graphs, * *p*-value < 0.05; ** *p*-value < 0.01; and *** *p*-value < 0.001 by Student’s *t*-test.

Whilst examining whether the 3D patient-derived CRLM model could be considered a valid in vitro test for drug screening, cytotoxicity, and prognosis, studies on the effect of 5-FU and FOLFIRI treatment when used on CRC cells cultured in 3D patient-derived scaffolds were performed. Repopulated CRLM and HL scaffolds and HT-29 grown in 2D were treated for three consecutive days with the experimentally-derived IC50 5-FU or FOLFIRI determined in 2D conditions (Figure 6A,C) (respectively, 1.3 and 1.01 µM, Supplementary Figure S7). No change in cell viability was detected in HT-29 cells when cultured in patient-derived scaffolds in the presence of 5-FU, with treated repopulated CRLM and HL scaffolds showing similar proliferation trends compared to non-treated counterparts (Figure 6A). The Caspase-3/Ki-67-positive cell ratio was similar between the different recellularized scaffolds, with a very low amount of Caspase-3-positive cells (Figure 6B,D). The complex 3D environment provided by the patient-derived scaffolds seemed to reduce the cell sensitivity to drugs (both 5-FU and FOLFIRI). In fact, CRC cells showed a comparable cellular turnover (Caspase-3/Ki-67-positive cell ratio) between treated and untreated scaffolds. Interestingly, by increasing the drug cytotoxicity using FOLFIRI, we found that the response of the CRC cell line to chemotherapy was scaffold-dependent. HT-29 cells grown in HL scaffolds exhibited a significant reduction in cell proliferation compared to untreated scaffolds (day 7 *p*-value = 0.002 and day 8 *p*-value = 0.023) (Figure 6C). In contrast, CRLM-repopulated scaffolds displayed an unaffected proliferation rate compared to untreated samples (Figure 6C). In parallel, HT-29 cells treated with 5-FU or FOLFIRI showed a significantly reduced proliferation rate compared with untreated cultures (5-FU, day 7 *p*-value = 0.004 and day 8 *p*-value = 0.0033; FOLFIRI, day 6 *p*-value = 0.005, day 7 *p*-value < 0.0001, and day 8 *p*-value < 0.0001) (Figure 6A,C).

## 3. Discussion

Metastasis formation is a multi-step process in which cancer cells escape from the primary tumor and survive in the circulation to seed and colonize distant sites [19]. Recent studies demonstrated that the behavior of metastases is strongly affected by the tissue-specific microenvironments they inhabit [8]. Once circulating, cancer cells colonize the metastatic site and their subsequent growth depends on the compatibility of the cancer cells—the “seed”—with the “soil” that they encounter in the new organ [20]. The results from this study show that it is possible to obtain a 3D in vitro culture system that closely mimics the phenotypical and biological properties of the in vivo metastatic microenvironment. The novelty of our model is the use of organ-specific ECM scaffolds for developing real 3D constructs directly derived from patients’ tissue samples using decellularization. This method generates biomimetic scaffolds without altering the biological and topological properties of the original ECM (no ECM digestion or plate coating is used in our method). In particular, we have demonstrated that *(i)* the decellularization of CRLM tissues produces scaffolds that recapitulate the histological, biochemical, and ultrastructural properties of the tissue of origin; *(ii)* CRLM scaffolds support CRC cell adhesion and influence proliferation, apoptosis, and migration in a tissue-specific manner; *(iii*) tissue-specific scaffolds stimulate distinct gene expression patterns in CRC cells cultured in distinct 3D environments; and *(iv)* CRC cells respond differently to standard chemotherapy when cultured in organotypic scaffolds compared to 2D conditions.

The decellularization method used in our study successfully eliminated the cellular compartment from the tissue, while preserving the ECM. Decellularized CRLM scaffolds recapitulated the histopathological architecture of pre-DET CRLM, such as the overexpression of neutral and acid mucins in comparison with HL [21]. Using non-invasive bioluminescence imaging to model CRC cell behavior in liver metastasis in vitro, we demonstrated that tumor-derived scaffolds (CRC and CRLM) supported a more proliferative phenotype compared to the respective normal counterpart, with seeded CRC scaffolds showing an even higher proliferation rate compared to CRLM scaffolds. These data were consistent with the clinical observation, for which an inverse relationship between CRC progression and tumor proliferative activity was reported [22]. In addition, the proliferation rate of CRC cells within the 3D scaffolds was substantially lower and more aligned with that of cells transplanted in vivo as xenografts, which serves as the current preclinical gold standard for assessing drug efficacy. Further analysis demonstrated that the difference in proliferation rates between cells cultured in CRC and CRLM scaffolds was not due to an increase in apoptosis in the CRLM scaffolds. 

The cell migration capability was also analyzed in patient-derived scaffolds by measuring bioluminescence at the far end of scaffolds and after a short culture period to minimize the contribution from cell proliferation [23,24]. We identified a significantly enhanced migration and invasion capability in CRC cells grown in CRLM scaffolds, compared with the healthy counterpart. Liver stroma is a dense network of sinusoidal microvessels, immersed in the space of Disse and mostly composed of reticular collagen, which are characterized by a discontinuous endothelial cell lining, leaving the underlying ECM components directly accessible to metastatic cells. It has been demonstrated that the in vitro selective blocking of α2β1-integrin, a specific receptor for reticular collagen on many tumor cells, causes cell migration and extravasation arrest, underlining the importance of cell interactions with the reticular collagen-rich fibers in the liver stroma [18]. In our study, we observed an increased abundance of reticular collagen in CRLM stroma compared with the HL counterpart that could partially explain the increased migration ability of CRC cells observed in CRLM scaffolds. The increase in the cell invasion and migration ability has been associated with tumor progression and identified by molecular, phenotypic, and behavioral changes called epithelial to mesenchymal transition. EMT is associated with a down-regulation of E-cadherin and an over-expression of Vimentin, SNAI-1, and ZEB-1 [25,26]. In many epithelial tumors, including CRC [27], the loss of E-cadherin leads to the loss of epithelial differentiation and the acquisition of a motile and invasive phenotype [28]. Up-regulation of Vimentin modulates the cytoskeletal organization and focal adhesion turnover, while SNAI-1 and ZEB-1 up-regulation drives EMT and inhibits apical–basal polarity components and cell–cell adhesion proteins such as E-cadherin [29]. In our study, a reduced E-cadherin expression coupled with an over-expression of Vimentin was observed in recellularized CRLM scaffolds. In addition, we were able to detect the known *SNAI-1*-induced over-expression of the *ZEB-1* gene, mainly when CRC cell lines were cultured in CRLM scaffolds, without any exogenous stimulation. The different results observed for EMT-related genes and protein expression between the two cell lines could be due to the fact that HCT-116 is a highly aggressive cell line with little or no capacity to differentiate (high stemness), while HT-29 has an intermediate capacity to differentiate into enterocytes and mucin-expressing lineages (low stemness) [30,31]. Based on this, we hypothesized that cells could respond differently to 3D patient-derived scaffold stimuli, based on their differentiation state. Taken together, these data confirmed that changes in cell behavior and phenotype were not only due to a switch from 2D to 3D culture conditions, but were the result of using specific organotypic patient-derived scaffolds. Conventional 2D cell culture systems are extensively used in in vitro drug screening applications. However, these culture conditions do not mimic the in vivo 3D cell-tissue organization and are lacking biochemical cues, and cell–cell and cell–ECM interactions [32,33,34,35,36]. In this study, genetic analysis showed that cells cultured in 3D patient-derived scaffolds had an organotypic-specific gene expression profile compared to 2D conventional cell culture systems, with the most highly represented biological processes involving the cellular response to stress, metabolic processes, the regulation of vesicle fusion, and the response to the oxygen level and starvation. On the other hand, HT-29-repopulated CRLM scaffolds exhibited a general down-regulation of genes involved in cell cycle regulation, DNA replication, and the regulation of transcription events fundamental in the G1/S transition of mitosis when compared to HT-29 cells grown in 2D. These results suggest a reduced uncontrolled proliferation in 3D cultured cells, possibly in favor of other pathways, to adapt to the surrounding environment. 

Demethylation and deacetylation processes, which are part of epigenetic regulation of the genome, were strongly activated in repopulated CRLM scaffolds. The global loss of DNA methylation is a well-established molecular hallmark of cancer cells [37]. DNA methylation appears to regulate metastasis initiation or metastasis progression genes that promote events such as angiogenesis, EMT, migration, invasion, and extravasation [38]. In particular, the loss of E-cadherin, a hallmark of EMT [39] and an important event that also occurred in our recellularized CRLM scaffolds, is mainly related to DNA methylation [40]. These epigenetic events were better represented when cells were cultured in decellularized scaffolds compared to 2D conditions, supporting the superiority of the 3D culture model in mimicking cancer in vitro. Moreover, decellularized HL did not influence cell DNA modifications to the same extent as CRLM scaffolds, further supporting the concept of microenvironment specificity and importance. Finally, we demonstrated that the cellular profile in 3D cultures closely resembles that of the in vivo tumor environment more than conventional 2D cultures, showing the activation of pathways involved in complex cell–cell and cell–ECM contact, hypoxia, and cell cycle heterogeneity. This was assessed by analyzing the transcriptomic profile of patient-matched CRC and CRLM tissue samples [41] and comparing this data with that of HT-29-repopulated CRLM scaffolds or 2D cultures. Specific transcriptomic signatures shared by recellularized CRLM scaffolds and human CRC and CRLM tissue samples included the response to hypoxia, EMT induction, and metabolic deregulation, revealing an overlap between our organotypic 3D culture model and the tumor cell profile in vivo.

Several studies have shown that the protein expression, gene expression, migration, morphology, proliferation, viability, organization, and drug response differ between 2D and 3D cancer models [35,42]. Given that CRC cells cultured in patient-derived scaffolds displayed cell behavior and gene expression closer to the in vivo metastatic environment, we expected to find that the 3D culture could influence the response to chemotherapy. Our results demonstrate that the response of 3D-cultured CRC cells to 5-FU and FOLFIRI administration was affected by the complex microenvironment provided by the tissue-specific decellularized scaffolds. HT-29 cells grown in CRLM scaffolds were more resistant to treatment with 5-FU and FOLFIRI at a conventional IC_50_ concentration (as determined in 2D cultures). This indicates that the 3D culture system better mimics the tumor microenvironment compared to 2D cultures, as tumor cells in vivo are usually non-responsive to chemotherapy agents used at a concentration effective in 2D cultures. These data are consistent with previous findings which demonstrated that 3D culture models lead to an up-regulation of proteins related to multi-drug resistance by mimicking characteristics of the in vivo tumor tissue, namely hypoxia [43], a low nutrient supply [7], and a low pH [44]. Further experiments will be necessary to address patient-to-patient and ECM areas’ heterogeneity and how they can impact cell behaviour and the response to treatment in our 3D model. Although a certain level of variability in cell behavior among constructs derived from the same tissue type is a limitation of our model, our data suggest that this culture system could be considered a suitable tool for in vitro mimicking of the liver metastatic microenvironment.

## 4. Materials and Methods

### 4.1. Patients

Five same-patient healthy colon mucosa (HC), primary colorectal cancer (CRC), healthy liver (HL), and colorectal cancer metastatic liver (CRLM) and 13 matched HL and CRLM tissue samples were collected from patients who underwent curative-intent surgery between January 2016 and July 2019 at the First surgery clinic (Department of Surgery, Oncology and Gastroenterology, University of Padua, Padua, Italy) and General Surgery Unit, S. Antonio Hospital (Padua, Itlay), or from patients who underwent partial hepatectomy as part of the treatment regime from November 2017 to November 2018 at King’s College Hospital (London, UK) (Table 1). This study was conducted according to the Declaration of Helsinki principles, written informed consent was obtained from the individual patients, and the ethics committees of institutions approved the protocol (Azienda Ospedaliera di Padova Ethical Committee Approved Protocol Number: P448 and King’s College Hospital Health Research Authority approval Research Ethics Committee ref: 17/NE/0340). All of the patients enrolled fulfilled the inclusion criteria of histologically-confirmed primary adenocarcinoma of the colon and or synchronous/metachronous liver metastasis, age >18 years. Patients with a known history of hereditary colorectal cancer and those that underwent neoadjuvant treatments were excluded. Eighteen CRLM and five CRC patients were enrolled. For tissue decellularization evaluation, proliferation, and migration analysis, EMT induction analysis- and gene expression analysis-matched healthy and tumor samples were used. For drug treatment analysis, matched and un-matched healthy and tumor samples were used.

### 4.2. Tissue Decellularization

CRC and CRLM tissues were obtained at the edge of infiltrating neoplasia. HC specimens encompassed the luminal surface, mucosa, and submucosa and were obtained more than 10 cm away from the primary CRC. HL samples were collected at least 0.5 cm away from the metastatic edge. All surgically collected specimens were kept in cold and sterile phosphate buffered saline (PBS) for no longer than 2 h before processing. HC, CRC, HL, and CRLM tissues destined for the pre-decellularization group were rinsed with sterile PBS and subsequently processed according to specific methodologies. Samples destined for decellularization following the detergent-enzimatic treatment were processed as described in [16] and in the supplementary methods.

### 4.3. DNA Isolation and Quantification

To assess the total DNA content within pre-decellularization HL and CRLM samples and compare the values with their DET counterparts, 20 mg of specimens was processed using the DNeasy Blood&Tissue kit (Qiagen, Hilden, Germany). DNA samples were quantified on a ND-1000 spectrophotometer (NanoDrop Technologies, Waltham, MA, USA).

### 4.4. Immunohistochemistry and Immunofluorescence

Formalin-fixed paraffin sections, 8 µm thick, were stained with Haematoxylin & Eosin (H&E; Bio Optica, Milan, Italy), Masson’s trichrome (aniline blue kit; Bio Optica), Alcian blue stain (pH 2.5 kit; Bio Optica), Van Gieson trichrome (Bio Optica), Silver Stain (Bio Optica), Periodic Acid Schiff (PAS; Bio Optica), Laminin (Sigma-Aldrich, St. Louis, MO, USA), HIF-1alpha, Collagen IV (Dako), Ki-67 (Abcam), E-cadherin (Abcam), activated Caspase-3 (Cell Signalling), and Vimentin (Abcam). Immunohistochemical staining was automatically performed using the Bond Polymer Refine Detection kit (Leica Biosystems, Wetzlar, Germany) in the BOND-MAX system (Leica Biosystems). Immunofluorescence was performed using previously published protocols [16] and as specified in the supplementary methods. Antibodies and dilutions used are shown in Supplementary Table S3. Control immunofluorescence stainings were performed on HT-29 and HCT-116 cells grown in 2D (respectively, Supplementary Figures S3 and S4).

### 4.5. Lentiviral Transduction of CRC Cells and FACS Sorting

The lentiviral vector pHIV-Luc-ZsGreen was a gift from Dr. Bryan Welm (Department of Surgery, University of Utah, purchased through Addgene Inc. MA, USA, Plasmid #39196) and was used to generate lentivirus expressing both ZsGreen fluorescent protein and firefly luciferase via an internal ribosome entry site under the EF1-alpha promoter. The transduction protocol is reported in the supplementary methods.

### 4.6. Scanning Electron Microscopy Analysis (SEM)

Samples were cut into segments of approximately 0.5 cm^2^ and fixed with 3% glutaraldehyde in 0.1 M PBS. After fixation and washing with 1X PBS, specimens were dehydrated in a graded ethanol-water series from 15% to 100% ethanol, critical point-dried using CO_2_, and finally mounted on aluminum stubs using sticky carbon taps. Samples were mounted and coated with a thin layer of Au/Pd (approximately 2 nm thick) using a Gatan ion beam coater. Images were recorded with a JEOL JSM 6490 scanning electron microscope (North Billerica, MA, USA).

### 4.7. Second Harmonic Generation Analysis (SHG)

SHG analysis was done using a custom-built multimodal microscope, described in detail elsewhere [45] and in the supplementary methods.

### 4.8. Recellularization of Decellularized Scaffolds and Cell Seeding in 3D-Bioprintable Ink

HC-, CRC-, HL-, and CRLM-decellularized matrices were incubated overnight with growth medium containing primocin antibiotic (InvivoGen) at 37 °C. In order to normalize intra-sample variability, scaffolds were cut into comparable dimensions before seeding. All matrices were then injected with 2.5 x 10^5^ HT-29 ZsGreen/Luc+ or HCT-116 cells, resuspended in 10 μL of Collagen I (diluted 2:3 with RPMI-1640), using a 30G syringe needle in a 24-well plate. In parallel, a drop of 100µL of 3D-bioprintable ink (Bioink—CELLINK Company, Sweden) was mixed with 2.5 × 10^5^ HT-29 ZsGreen/Luc+ cells and deposited in a 24-well plate. Samples were incubated for 6 h in a humidified incubator at 37 °C and 5% CO_2_. Complete medium (supplementary methods) was added and changed every other day. Recellularized samples used for RNA extraction were immediately frozen in liquid nitrogen at the end of the experiment and stored at −80 °C. Recellularized samples were either formalin-fixed and paraffin-embedded or fixed in 4% PFA and OCT- or gelatin-sucrose-embedded. 

### 4.9. Bioluminescent Image Analysis

The luciferase gene in the lentivirus allows the visualization of cells by bioluminescence in the presence of D-luciferin substrate (Cayman Chemicals, UK). To standardize the bioluminescence detection, HT-29 cells were seeded in 24-multiwell dishes and incubated with increasing concentrations of D-Luciferin (ranging from 1 to 20 µg/mL). A time course analysis of bioluminescence was performed at all D-Luciferin concentrations using medium alone as the negative control. The bioluminescence signal was measured every 5 min, for up to 60 min, after D-Luciferin addition, using the IVIS Lumina III In Vivo Imaging System (IVIS) and the Living Image 3.2 software (Caliper Life Sciences, Waltham, MA, USA). For further details, see the supplementary methods.

### 4.10. Cell Migration

An investigation of cell migration in 3D scaffolds was performed by injecting HT-29 cells into the left side of the scaffolds and culturing them for 4 days. Bioluminescence was detected every 24 h. Quantitative analyses of migration were obtained by measuring the bioluminescence in four ROIs in sequence (from left to right) to identify the distance covered by cells within the scaffolds. The fold increase in average radiance at the far right-end of the scaffolds (ROI d) at day 4 with respect to day 0 was determined as the maximum distance travelled by the cells. Migration in seeded scaffolds was than qualitatively confirmed by H&E staining.

### 4.11. RNA Extraction, Microarray, and qRT-PCR Analysis

Transcriptomic analysis was performed by comparing the global gene expression profiles of HT-29 cells grown on CRLM and HL scaffolds. Recellularized samples used for obtaining the gene expression profile were gently thawed in ice. Total RNA was extracted and quality checked, as reported in [16]. Microarray analyses were performed using the Clariom S Assay (ThermoFisher Scientific, Waltham, MA, USA), according to the manufacturer’s instructions, starting from 100 ng of total RNA. qRT-PCR analyses of Snai-1 and Zeb-1 genes were performed using specific TaqMan^®^ Gene Expression Assay 1× (Applied Biosystems), according to the manufacturer’s instructions, starting from 1 µg of total RNA. See the supplementary methods for further details.

### 4.12. 5-Fluorouracil and Folfiri Treatment and Cytotoxicity Assay

For the 5-Fluorouracil (5-FU) and 5-FU combined with Irinotecan (FOLFIRI) treatment in the 3D setting, CRLM and HL scaffolds were seeded with 2.5 × 10^5^ CRC cells in 24-well plates. Five days post-seeding, cells were treated with 1 µM 5-FU or FOLFIRI (both 5-FU and Irinotecan at 1 µM) for 72 h. For the 5-FU treatment of the 2D culture, 5-FU or FOLFIRI was added after 5 days of culture. HT-29 cells were seeded at 2 × 10^4^ cells per well in 24-well plates. Cell viability was determined by bioluminescence reading with IVIS at day 0, 1, 3, 5, 6, 7, and 8, and luciferin was added fresh every time. During the 72 h of treatment, 5-FU or FOLFIRI was only added once (day 5).

### 4.13. Statistical Analysis

All graphs and statistical analyses were performed using GraphPad Prism Software (version 6, GraphPad Software, San Diego, CA, USA). Data are expressed as means ± S.E.M. For a comparison of coupled experimental groups, two-sided Student’s *t*-tests (for the parametric dataset) and the Mann–Whitney test (for the non-parametric dataset) were used. One-way ANOVA with Bonferroni’s post-test (for the parametric dataset) and the Kruskal–Wallis test with Dunn’s post-test (for the non-parametric dataset), were performed for multiple comparisons. A *p*-value < 0.05 was considered statistically significant (* *p*-value < 0.05; ** *p*-value < 0.01; *** *p*-value < 0.001).

## 5. Conclusions

The lack of a stromal cell component represents a limitation of our culture systems, especially when aiming to study tumor cell–stroma interactions. To address this issue, and to improve the sophistication of the 3D patient-derived culture model, liver stromal cells and a dynamic perfusion system need to be added. However, the present culture system provides an engineered model of CRLM which recapitulates CRLM architectural and biochemical cues, and which could represent a powerful tool for capturing the relevant cell–microenvironment interactions in metastatic cancer in a more tissue-specific manner compared to conventional 2D culture systems. 

In conclusion, we have established a physiologically-relevant 3D tissue culture model which is able to mimic in vivo features of CRLM, such as the proliferation, migration, and chemotherapeutic drug response of CRC cells in liver metastatic tissue and, as such, represents a powerful tool for investigating CRLM biology in vitro.

## Figures and Tables

**Table 1:** Clinic-pathological characteristics of CRLM and CRC patients.

Characteristic	Parameter	CRLM Patients (*n* = 18)
Age	Median (range), yrs	65 (45–87)
Sex	Male Female	7 (39.9%) 11 (61.1%)
cTNM	IV	18 (100%)
Metastasis onset	Synchronous Metachronous Not-available	10 (55.6%) 4 (22.2%) 4 (22.2%)
**Characteristic**	**Parameter**	**CRC Patients** **(*n* = 5)**
Age	Median (range), yrs	62 (54–81)
Sex	Male Female	4 (80%) 1 (20%)
Grade	1 2 3	1 (20%) 4 (80%) 0 (0%)
cTNM	I II III IV	0 (0%) 0 (0%) 1 (20%) 4 (80%)

CRLM: colorectal cancer liver metastases; CRC: colorectal cancer; Yrs: years; cTNM: clinical Tumor Node Metastasis stage.

## Supplementary Materials

The following are available online at https://www.mdpi.com/2072-6694/12/2/364/s1. Figure S1: Histological and immunohistochemical evaluation of collagen content before and after tissue decellularization. Figure S2: Transduction of HT-29 cells with pHIV-Luc-ZsGreen lentiviral vector. (A) Vector– panel: bright field image of non-transduced HT-29 cells. Figure S3 Characterisation of HT-29 cells in 2D cultures. Figure S4: Characterization of HCT-116 recellularized CRC and CRLM scaffolds, after 10 days of culture, using immunofluorescence and gene expression analysis. Figure S5: HT-29 ZsGreen/Luc+ cells seeded in the 3D-bioprintable ink. Figure S6: Transcriptomic analysis of recellularized HL and CRLM scaffolds and Luc-ZsGreen+HT-29 cells cultured in 2D. Figure S7: Calculation of IC50 concentration for HT-29 in 2D culture conditions. Table S1: List of 190 differently expressed genes between recellularized CRLM scaffolds vs HT-29 grown in 2D. Table S2: List of 53 differently expressed genes between recellularized CRLM scaffolds vs recellularized HL scaffolds following Limma analysis. Table S3. List, host, dilution and manufactures information of the antibodies used for Immunofluorescence or immunohistochemistry analyses.
