# Supplementary material for: Patient-Derived Scaffolds of Colorectal Cancer Metastases as an Organotypic 3D Model of the Liver Metastatic Microenvironment
Source: Cancers (Basel). 2020 Feb 5;12(2):364. doi: 10.3390/cancers12020364 (PMC7072130; doi:10.3390/cancers12020364)
Supplement: Supplementary file 1 [file cancers-12-00364-s001.zip › cancers-673290-suppl.pdf]

Supplementary Materials:

Patient-Derived Scaffolds of Colorectal Cancer Metastases as an Organotypic 3D Model of the Liver Metastatic Microenvironment

Edoardo D'Angelo, Dipa Natarajan, Francesca Sensi, Omolola Ajayi, Matteo Fassan, Enzo Mammano, Pierluigi Pilati, Piero Pavan, Silvia Bresolin, Melissa Preziosi, Rosa Miquel, Yoh Zen, Shilpa Chokshi, Krishna Menon, Nigel Heaton, Gaya Spolverato, Martina Piccoli, Roger Williams, Luca Urbani and Marco Agostini

Supplementary Materials

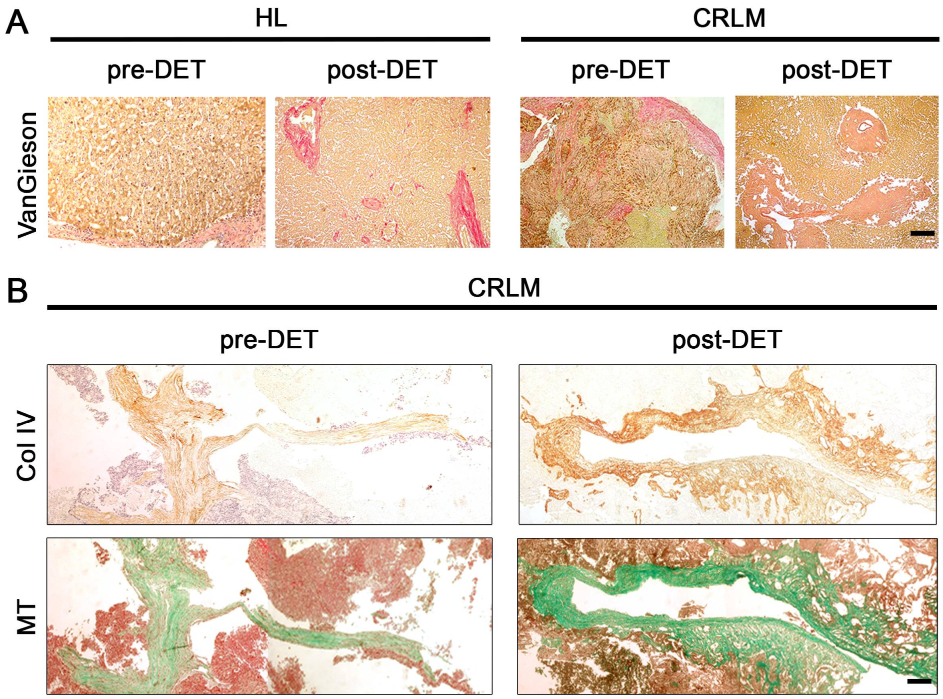

Figure S1. Histological and immunohistochemical evaluation of collagen content before and after tissue decellularization. **(A)** VanGieson's stain of HL and CRLM samples before (pre-DET) and after (post-DET) decellularization confirmed the preservation of collagen structures around vessel walls for both HL and CRLM scaffolds. **(B)** Anti-collagen IV immunostaining and Masson's Trichrome (MT) in pre- and post-DET CRLM tissues. Scale bar: 100μm.

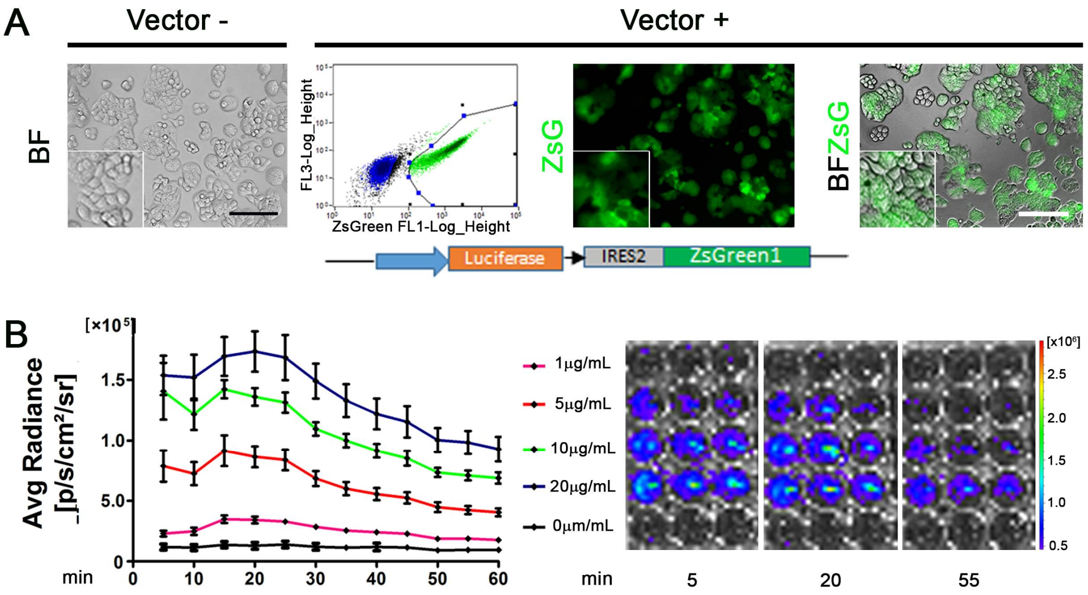

Figure S2. Transduction of HT-29 cells with pHIV-Luc-ZsGreen lentiviral vector. **(A)** Vector– panel: bright field image of non-transduced HT-29 cells. Vector+ panel: FACS image show separation of a pure ZsGreen+ population of cells after transduction with an MOI of 1-2. Fluorescence images of transduced HT-29 cells show that transduction does not alter cell shape and morphology; Scale bars: 100 μm. **(B)** Optimization of bioluminescent signal acquisition measured on cells incubated with increasing concentration of D-luciferin (range 1-20μg/ml) and signal acquisition times from minute 5 to 60, every 5 minutes. Left panel, line graph representing the bioluminescence average radiance for all concentrations. Right panel, a representative image of IVIS pictures after 5, 20 and 55 minutes from D-luciferin incubation.

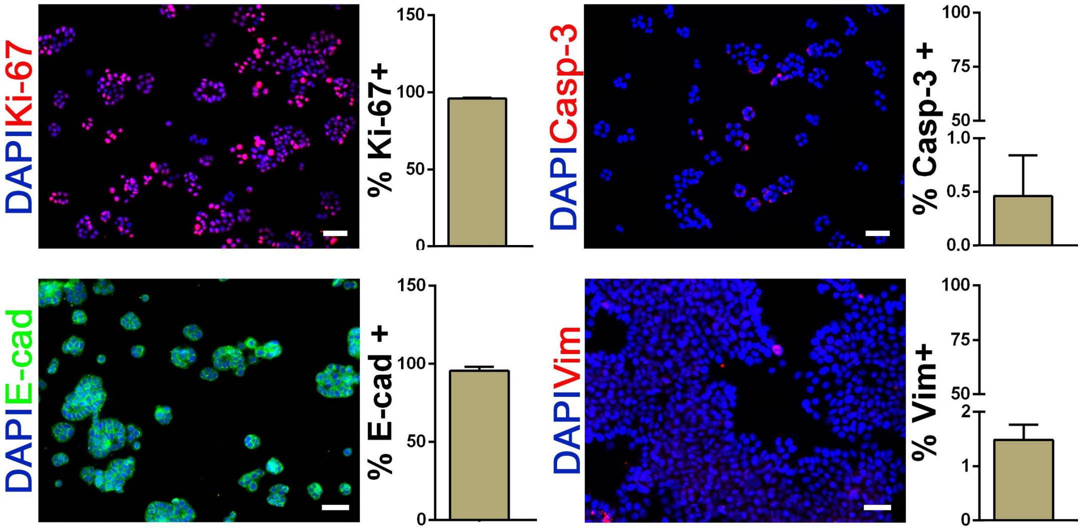

Figure S3. Characterisation of HT-29 cells in 2D cultures. Immunofluorescence and relative counts of HT-29 cells cultured in conventional 2D conditions showing expression of Ki-67+, Activated Caspase-3+, Vimentin+ (all in red) and E-cadherin+ (green). Nuclei are counterstained with DAPI (blue). Scale bar: 50μm.

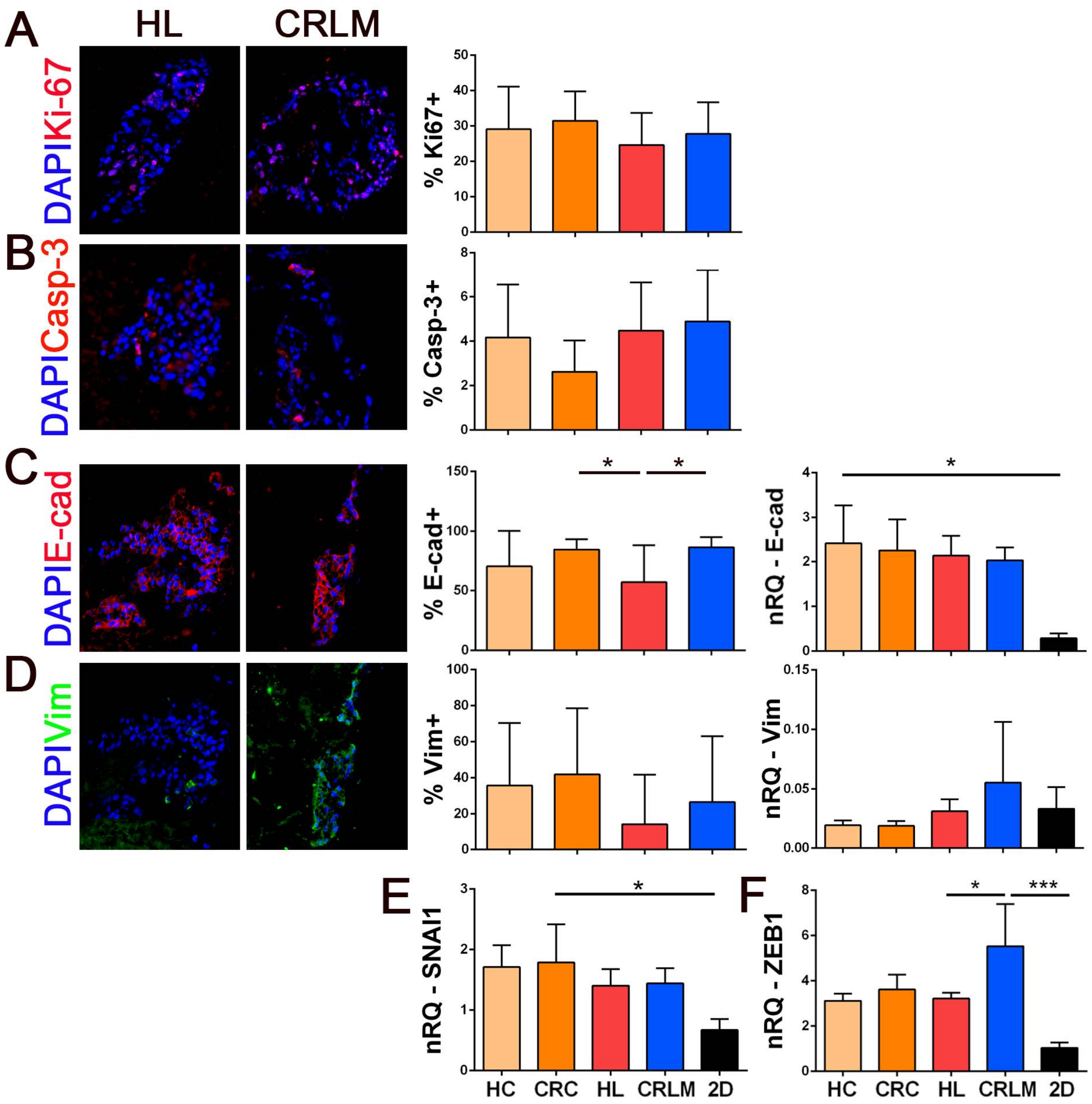

Figure S4. Characterization of HCT-116 recellularized CRC and CRLM scaffolds, after 10 days of culture, using immunofluorescence and gene expression analysis. Immunofluorescent images and quantifications at day 10 of **(A)** Ki-67+, **(B)** Activated Caspase-3+, **(C)** E-cadherin+ (red), **(D)** Vimentin+ cells (green) and **(E)** Gene expression level of SNAI-1 in repopulated HC, CRC, HL, CRLM and conventional plastic plate (2D). **(F)** Gene expression level of ZEB-1 in repopulated HC, CRC, HL, CRLM and conventional plastic plate (2D). Scale bar=50μm for immunofluorescence data. nRQ=normalized Relative Quantity for qRT-PCR data. Graph bars, *p-value<0.05; **p-value<0.01 and ***p-value<0.001 by 1 way ANOVA test following Tukey's Multiple Comparison Test.

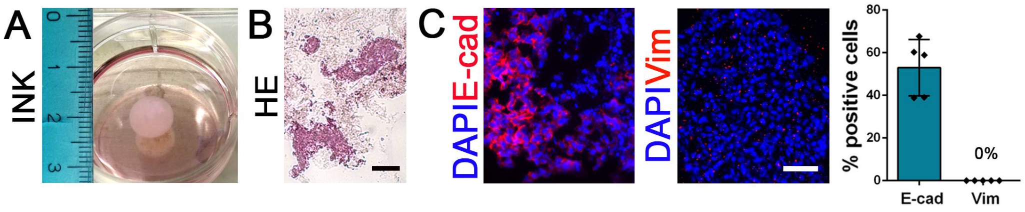

Figure S5. HT-29 ZsGreen/Luc+ cells seeded in the 3D-bioprintable ink. **(A)** gross appearance of 3D construct in the cell culture plate; **(B)** H&E, **(C)** representative images and relative counts of E-cadherin+ and Vimentin+ cells after 10 days of culture. Scale bar=50μm.

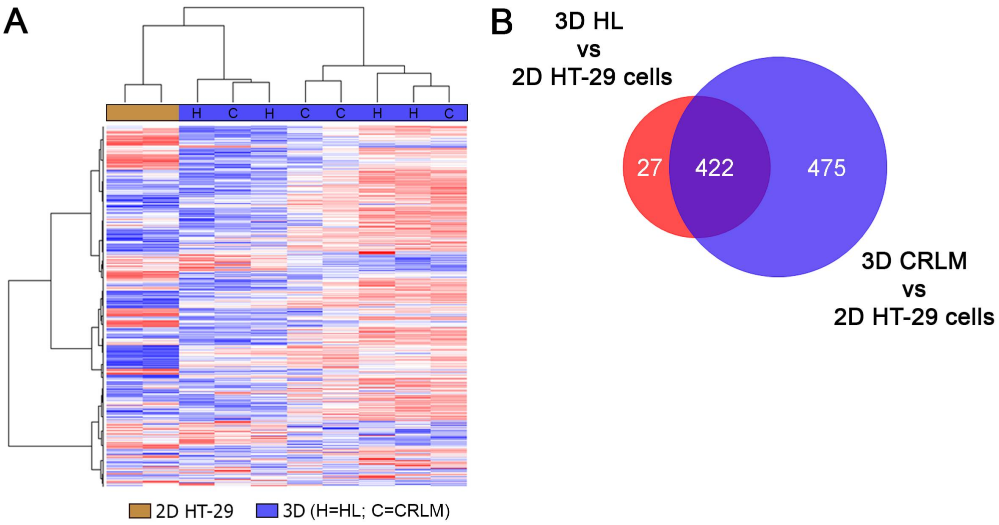

Figure S6. Transcriptomic analysis of recellularized HL and CRLM scaffolds and Luc-ZsGreen+HT-29 cells cultured in 2D. **(A)** Unsupervised hierarchical clustering analysis of recellularized HL and CRLM scaffolds in respect to Luc-ZsGreen+HT-29 cells grown in 2D. **(B)** SAM analysis of differently expressed genes (DEG) between recellularized HL or CRLM and Luc-ZsGreen+HT-29 cells cultured in 2D CRLM scaffolds.

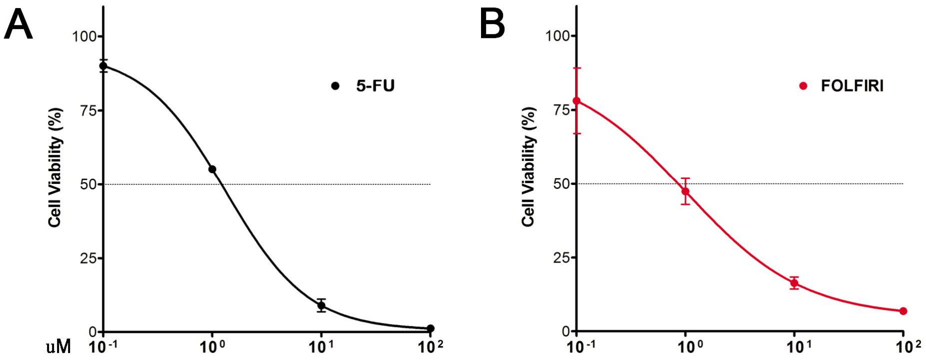

Figure S7. Calculation of IC50 concentration for HT-29 in 2D culture conditions for **(A)** 5-FU and **(B)** FOLFIRI.

Tables SI and S2 can be found in the Separately Excel file

Supplementary Table S1: List of 190 differently expressed genes between recellularized CRLM scaffolds vs HT-29 grown in 2D belonging to "EPITHELIAL_MESENCHYMAL_TRANSITION" sub-group of GSEA Hallmark dataset. Running ES = Running Enrichment score.

Supplementary Table S2: List of 53 differently expressed genes between recellularized CRLM scaffolds vs recellularized HL scaffolds following Limma analysis. For 10 out of 53 of them references regarding their involvement in invasion, migration and epithelial to mesenchymal transition induction were provided. ID=Specific probe associated to the gene in the Clariom S genechip (Thermofisher); Normal Avg = Normalized Averaged.

© 2020 by the authors. Licensee MDPI, Basel, Switzerland. This article is an open access article distributed under the terms and conditions of the Creative Commons Attribution (CC BY) license (http://creativecommons.org/licenses/by/4.0/).
